# Four key questions about metformin and cancer

**DOI:** 10.1186/s12915-014-0085-1

**Published:** 2014-10-24

**Authors:** Navdeep Chandel

**Affiliations:** Section of Pulmonary and Critical Care Medicine, Department of Medicine, Feinberg School of Medicine, Northwestern University, Chicago, IL 60611 USA

Navdeep Chandel took a BA in mathematics and a PhD in cell physiology at University of Chicago, where he also did his post-doctoral work, on cytochrome c oxidase. The recognition of the role of mitochondria in activating apoptosis and hypoxic responses led him to his present research focus on mitochondria as signaling organelles at Northwestern University, where he is a Professor of Medicine and Cell Biology. What follows is the edited and updated transcript of an interview he gave at the Biomed Central conference on Metabolism, Diet and Disease in May [1], where the topic was cancer and metabolism and he spoke on the contentions surrounding the issue of whether and how the antidiabetic drug metformin acts to inhibit tumor growth.

## There have been very high hopes for using metformin and other biguanides against cancer. How is that class of drug thought to work in cancer?

There are two ideas about how metformin works. Epidemiological studies on people taking metformin for diabetes suggested that they had a lower incidence of cancer, and this was in multiple types of cancers. One obvious effect of metformin in people who have high levels of insulin is that their insulin levels come down. That now has an effect on the cancer cells.

What is that effect? Well, one big effect is because insulin is a mitogen. Insulin and insulin growth factors can activate a signaling kinase pathway that depends on the PI3-kinase pathway. We know that is the growth pathway. The data are very clear: in cancer and throughout the metazoans, that’s how we grow.

So imagine that somebody has high levels of insulin, and that metformin decreases those insulin levels. The way metformin does that is it gets into the liver and prevents gluconeogenesis from occurring, primarily. So your liver is not putting out as much glucose, so your insulin levels decrease. As your insulin levels decrease, therefore, you have less mitogenic stimulation on the cancer cells and this is one way, perhaps, you can reduce the tumor.

The second effect - and the two effects are not necessarily mutually exclusive - is that the metformin actually gets into the cancer cells: that there are transporters that allow metformin to accumulate in cancer cells. And then in the cancer cells it would hit some target and that would then stop the cells from proliferating. Now, what that target is has been elusive. People have been trying to figure this out. We recently looked at the literature and decided that the most promising target in cancer cells has been mitochondrial complex I. I worked with my group to come up with some ways to test whether complex I inhibition was necessary for the anti-tumor effects. The data suggested they were [2].

So I think it’s possible there are two mechanisms. One is metformin hits the insulin and decreases insulin levels, so you have less of a mitogenic stimulation to the PI3-kinase pathway. The other one is - it gets eventually into the mitochondria to inhibit complex I. There could be other targets in mitochondria, including one that Gerry Shulman’s lab has proposed, which is glycerol-3-phosphate dehydrogenase [3]. This applies more to the effects of metformin on gluconeogenesis. Whether that’s related to anti-cancer effects, we don’t know. But basically, you could have these two different anti-tumor effects for metformin: indirect, through insulin, and direct through complex I or perhaps other targets in the mitochondria.

## Because of the interest in anti-cancer effects of metformin there have been clinical trials, but they’ve had inconclusive - or perhaps I should say inconsistent - results between one trial and another. It’s not clear that it actually works. Why not?

Michael Pollak makes a great point when he says the problem with metformin is it’s cheap, it’s widely available, it has a great safety profile, and anyone can use it.

## That’s a problem?

Yes, because what you have is a rush to do clinical trials because there is almost no barrier to entry. In fact, if you Google metformin as a cancer patient, you might find some data on it and you could say to your doctor, 'I want to be put on Metformin', and your doctor would have no reason to say no. As opposed to conventional drug development - this is done very carefully: they do toxicity studies; they carefully rationalize who should get the treatment. Here there’s no rationalization. It’s there for all comers, right? But of course if you don’t know the mechanism then it makes it very difficult to know who’s going to benefit.

So with the knowledge that we think we have now about metformin and how it may work by these two mechanisms - the insulin receptor-dependent mechanism and the one that goes through mitochondrial complex I - you can now take biopsies and actually characterize the tumor cells with these two rationales in mind.

You’d want to look at four parameters, I would say. One parameter is: are the tumor cells insulin receptor-positive or IGF - insulin growth factor receptor - positive? Second, does the patient have high levels of insulin? And then when they get on metformin, do those levels of insulin come down? What happens to the receptor - is it still there after metformin treatment?

The third parameter is critical to the cell autonomous function, where the question is whether the metformin would get into the cell and get access to the mitochondrial respiratory complex. You need the right transporters, in this case organic cation transporters. There are three of them. Again, with modern technology you can see - are the tumor cells positive for those transporters? If they are, then metformin can accumulate in the cell.

Then, fourth, you have to get into the mitochondria as well. You can look for metabolic signatures - as I call them, the ox-phos, the oxidative phosphorylation metabolic signatures. Some tumor cells might have a more robust ox-phos metabolic signature or maybe they’re not as dependent on glucose, they’re not as PET-positive for glucose.

So you can imagine now - if I was designing a clinical trial, I’d want to know the insulin levels, the insulin receptor, the organic cation transporters, and a signature of mitochondrial and glycolytic metabolic function. And then - let’s see what happens. If you have all four then you have multiple ways metformin could affect tumor growth.

I would start with that, but now we can also think of what rational therapeutic combinations would work. Because no one single agent works. Most people will try chemotherapy, and I can tell you it’s not clear whether chemotherapy is the best thing with metformin. Maybe it is initially. But a lot of the chemotherapeutic drugs work through reactive oxygen species (ROS). Metformin acting cell autonomously can either increase or decrease ROS: maybe metformin can in some cases mitigate the effects of chemotherapy by not allowing ROS generation, or it may in some cases increase ROS generation. So it gets very muddled. But what’s clear is that decreasing your ability to take up glucose, for example, would shut off or decrease glycolysis, and metformin would decrease ATP production from mitochondria. With the two together, there’s very little energy. Even a cancer cell can’t get around that, if you block both of the ways to generate energy.

This is why I always tell people at my own university - the clinicians - that it pays to know the mechanism because then we can perhaps advise them on rational ways to go about clinical trials. That’s a basic scientist speaking not a clinician! Perhaps a bit biased.

## From a practical point of view, what evidence is there for real effects *in vivo* of bigaunides, including metformin, on tumor growth?

In an experimental setting, in the laboratory setting, there have been quite a number of studies done in mouse models of cancer where you put the mouse on metformin and it diminishes tumor growth. We recently replaced mitochondrial respiratory complex I function in mice with a single yeast protein which can confer some of the complex I activities, but is resistant to metformin [3]. In those mice, the tumors still grew out in the presence of metformin, because they had, essentially, an intact protein to replace complex I activity.

So we think complex I is necessary for metformin’s anti-tumor effects *in vivo*. But there have been questions about whether the concentrations of metformin that people like myself have used in *in vivo* studies, or that Craig Thompson’s used - he had a beautiful paper six or seven years ago that got the field excited - whether they are clinically relevant concentrations. There are those who would argue that we’re using, in the mouse, supraphysiological levels of metformin. One thing that is missing is a pharmacokinetic analysis - perhaps some of us in the field should just go back and do some real pharmacokinetics on metformin at the concentrations that we’re delivering to the mice in the plasma, but more importantly the tumor. You know, grind up the tumor, figure out how much got in. To get some rough estimates.

Something that you have to remember: even if you start clinically with somewhere like 20 micromolar in the plasma, the way that metformin gets into the mitochondria is based on the mitochondrial membrane potential. So of that 20 micromolar in the plasma, the amount that would accumulate in the mitochondrial matrix would be almost 100 times more. Or 1,000 times more. That’s important because you go from 20 micromolar to 20 millimolar in the matrix, if it’s a thousand-fold increase. Because metformin - as very elegant work from Judy Hirst has pointed out - is a weak inhibitor of complex I - something like 20 millimolar, if I recall correctly from her talk at this meeting is the IC50 [4]. You need millimolar to get in, but because of the membrane potential - you can really, really enrich metformin. So when people say, 'OK, 20 micromolar, but you need 20 millimolar', we have a good answer to that, because of enrichment by the membrane potential.

Nevertheless, I think - again, because it’s a cheap drug, we’ve used it for years - no one’s really answered those questions: what is the clinical concentration? We use the anti-diabetic dose in patients for cancer therapy in the current clinical trials. Maybe we need to use higher doses than the anti-diabetic dosing for metformin as an anti-cancer agent. What’s interesting is that one of the cancers metformin works best in is colon cancer, both experimentally and epidemiologically. This makes good sense because metformin does accumulate at very high levels in the colon compared to other places.

So this is an important question and the debate continues. I think, in the end, there is a direct target in the tumor cell; it’s not all insulin and organismal metabolism that explains the epidemiology. As long as the tumor cell has the transporters, it can take it up, get it into the mitochondria, and then it can inhibit ATP or ROS production and make the cells anti-proliferative.
